# Arterial spin labeling MR imaging aids to identify cortical venous drainage of dural arteriovenous fistulas

**DOI:** 10.1097/MD.0000000000010697

**Published:** 2018-05-11

**Authors:** Ji Hee Kang, Tae Jin Yun, Jong Kook Rhim, Young Dae Cho, Dong Hyun Yoo, Roh-Eul Yoo, Koung Mi Kang, Seung Hong Choi, Ji-hoon Kim, Chul-Ho Sohn, Moon Hee Han

**Affiliations:** a Institute of Radiation Medicine, Seoul National University Medical Research Center; b Department of Radiology, Seoul National University Hospital, Seoul, Republic of Korea.

**Keywords:** arterial spin-labeling, arteriovenous fistula, cerebral blood flow, cortical venous drainage, MR perfusion imaging

## Abstract

Cortical venous drainage (CVD) increases the probability of intracranial hemorrhage and mortality rate of dural arteriovenous fistulas (DAVF). Although digital subtraction angiography (DSA) is the most accurate method to determine CVD in DAVFs, this modality has limitations due to its invasive nature and radiation issues. The purpose of this study was to evaluate the diagnostic utility of arterial spin-labeling perfusion-weighted images (ASL-PWI) to identify CVD in patients with DAVF.

The Institutional Review Board of our hospital approved this retrospective study. ASL-PWI features of 22 patients with DAVF were retrospectively reviewed for the presence of bright signal intensity in cortical veins and brain parenchyma. DAVF with bright signal intensity in cortical veins and/or brain parenchyma was regarded as having CVD. Using DSA as a reference standard, sensitivity, specificity, positive predictive value, and negative predictive value of ASL-PWI for detecting CVD were calculated.

Based on DSA features, 11 (11/22, 50%) patients were classified as having “aggressive” pattern with CVD. Eleven (11/22, 50%) patients also showed bright signal intensity in cortical veins (9/22, 41%) and/or brain parenchyma (4/22, 18%) on ASL-PWI. The 11 patients who had “Aggressive” pattern on DSA were the same 11 patients who were classified as having “aggressive” pattern on ASL-PWI. ASL-PWI showed perfect diagnostic performance for identifying CVD with sensitivity, specificity, positive predictive value, and negative predictive value of 100% for all.

Thus, ASL-PWI could be used as a noninvasive mean to predict the presence of CVD in patients with DAVFs. It has the potential as a screening tool to evaluate DAVF prior to invasive DSA.

## 1 Introduction

Dural arteriovenous fistula (DAVF) is a cerebral vascular malformation characterized by direct communication between dural arteries and dural sinuses or cortical veins.^[1,2]^ Cortical venous drainage (CVD) increases the probability of intracranial hemorrhage and mortality rate of patients with DAVF.^[1–3]^ Although multiple modalities such as CT and MR images with angiography can be used to diagnose DAVF, digital subtraction angiography (DSA) is the most accurate method to determine CVD in DAVFs.^[4]^ However, DSA has limitations due to its invasive nature and radiation issues.

Arterial spin-labeling perfusion-weighted image (ASL-PWI) is a noninvasive imaging technique that uses water as an endogenous contrast agent by magnetically labeling it with radiofrequency pulses.^[5,6]^ Previous studies have shown that ASL, as other perfusion imaging techniques, can be applied to various cerebrovascular diseases. It has been reported that ASL can depict areas of perfusion defect in acute stroke.^[7,8]^ ASL also can show perfusion change in patients with Moyamoya disease.^[9]^ In addition, ASL-PWI is useful for depicting draining veins as hyperintense venous signal in patients with DAVF.^[10–12]^ Recently, ASL-PWI has been incorporated as part of the cerebrovascular disease evaluation in our institution. With its increasing use, we have encountered DAVF patients with CVD showing characteristic bright signal in cortical veins or affected brain parenchyma on ASL-PWI images.

The purpose of this study was to investigate the diagnostic utility of ASL-PWI for identifying CVD in patients with DAVF.

## 2 Methods

This retrospective study was approved by the Institutional Review Board of our hospital. The requirement for informed consent was waived due to its retrospective nature.

### 2.1 Patients

After searching our radiology database between January 2012 and July 2015, a total of 78 patients with DAVF diagnosis based on DSA were found. Among these 78 patients, 30 patients who underwent ASL-PWI were included. The following exclusion criteria were used in this study: no available ASL-PWI images before completion of treatment (n = 3), ASL-PWI images of poor image quality due to inadequate acquisition times or artifacts (n = 3), or patients who had other cerebrovascular problems such as cerebral venous thrombosis (n = 2). Finally, 22 patients were included in this study, including 10 men and 12 women whose median age was 64 years (range, 20–82 years).

### 2.2 MR Imaging protocol

All patients underwent MRI with a 1.5 T unit (Signa HDTx; GE Medical Systems, Milwaukee, WI [n = 16]) or 3 T unit (Discovery 750; GE Medical Systems, Milwaukee, WI [n = 6]). MRI sequences consisted of T1-weighted image (T1WI), T2-weighted image (T2WI), fluid-attenuated inversion recovery (FLAIR), diffusion-weighted imaging (DWI) (b factors, 0 and 1000 mm/s^2^), and ASL-PWI. ASL-PWI scans were performed using a pseudocontinuous ASL-PWI pulse sequence. Signal intensity change between labeled and control images was fitted to a previously published model to obtain a quantitative perfusion map of cerebral blood flow.^[13]^

### 2.3 Image analysis

Two interventional neuroradiologists (JKR and YDC with 5 and more than 10 years of experiences, respectively) reviewed DSA images with consensus. The following angiographic findings were reviewed: location of fistula, feeding artery, draining vein, flow pattern in sinus, and the presence of CVD. Based on Cognard classification, angiographic features were classified into 2 groups: “benign” pattern in which CVD was absent (types I and IIa); and “aggressive” pattern in which CVD was present (types IIb, IIa+b, III, IV, and V).^[3]^ Two radiologists (JHK and TJY with 2 and more than 10 years of experiences, respectively) reviewed ASL-PWI images with consensus. The following ASL-PWI features were analyzed: the presence of bright signal intensity in dural sinuses, the presence of bright signal intensity in cortical veins, and the presence of bright signal intensity in brain parenchyma. Based on ASL-PWI features, DAVF were classified into 2 groups: “benign” pattern with neither finding the presence of bright signal intensity in cortical veins nor the presence of bright signal intensity in brain parenchyma; and “aggressive” pattern with the presence of bright signal intensity in cortical veins and/or the presence of bright signal intensity in brain parenchyma. To evaluate the location of bright signal intensity on ASL-PWI, a 3D localization tool available on the picture archiving and communication system was used.

### 2.4 Statistical analysis

All statistical analyses were performed using statistical software program MedCalc version 11.1.1.0 (MedCalc, Mariakerke, Belgium). Descriptive statistics are presented as number and percentage.

## 3 Results

Clinical information and imaging findings of these patients are summarized in Table 1.

**Table 1:**
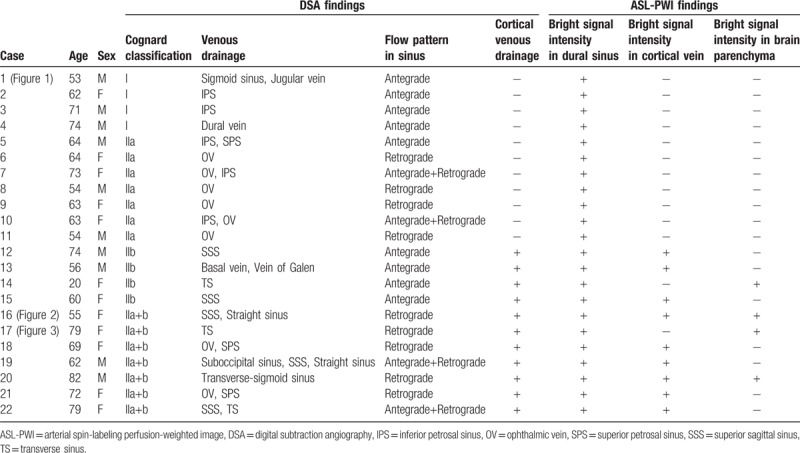
Clinical information and imaging findings of patients used in this study.

Based on DSA features, 11 (11/22, 50%) patients were classified into “aggressive” pattern with CVD. All patients (22/22, 100%) showed bright signal intensity in dural sinuses on ASL-PWI. Eleven (11/22, 50%) patients showed bright signal intensity in cortical veins (9/22, 41%) and/or brain parenchyma (4/22, 18%) on ASL-PWI. They were classified as having “aggressive” pattern, including 2 (9%) patients who showed both findings of bright signal intensities in cortical vein and brain parenchyma. The 11 patients who had “aggressive” pattern on DSA were the same 11 patients who were classified as having “aggressive” pattern on ASL-PWI.

The sensitivity, specificity, positive predictive value, and negative predictive value of ASL-PWI for differentiating between “benign” and “aggressive” patterns with respect to DSA features were all 100% (95% confidence intervals [CIs]: 72%–100%). In terms of sole finding of bright signal intensity in cortical vein, sensitivity, specificity, positive predictive value, and negative predictive value of ASL-PWI for differentiating between “benign” and “aggressive” patterns were 82% (9/11, [95%CI:48%,98%]), 100% (11/11, [95%CI:72%,100%]), 100% (9/9, [95%CI:66%,100%]), and 85% (11/13, [95%CI:85%,98%]), respectively.

**Figure 1:**
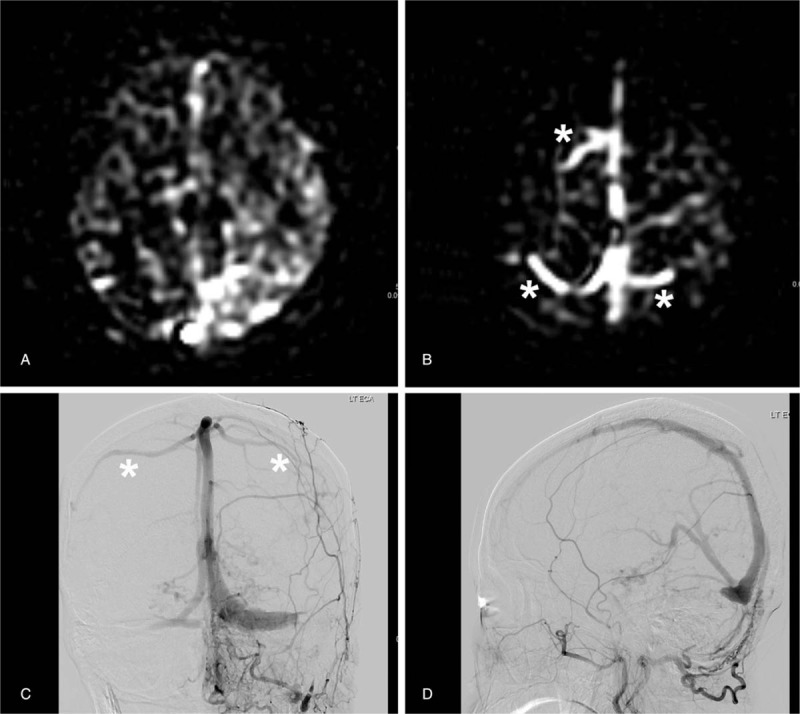
A 53-year-old man presented with left-sided tinnitus. (A) ASL-PWI shows bright signal intensity apparent in the left sigmoid sinus at distal to DAVF (arrow). However, bright signal intensity in the cortical vein is not definite on ASL-PWI. (B and C) DSA images reveal early hypervascular staining in the left sigmoid sinus and internal jugular vein (arrows) and DAVF (arrowheads). However, CVD is not definite on DSA. ASL-PWI = arterial spin-labeling perfusion-weighted image, CVD = cortical venous drainage, DAVF = dural arteriovenous fistula, DSA = digital subtraction angiography.

**Figure 2:**
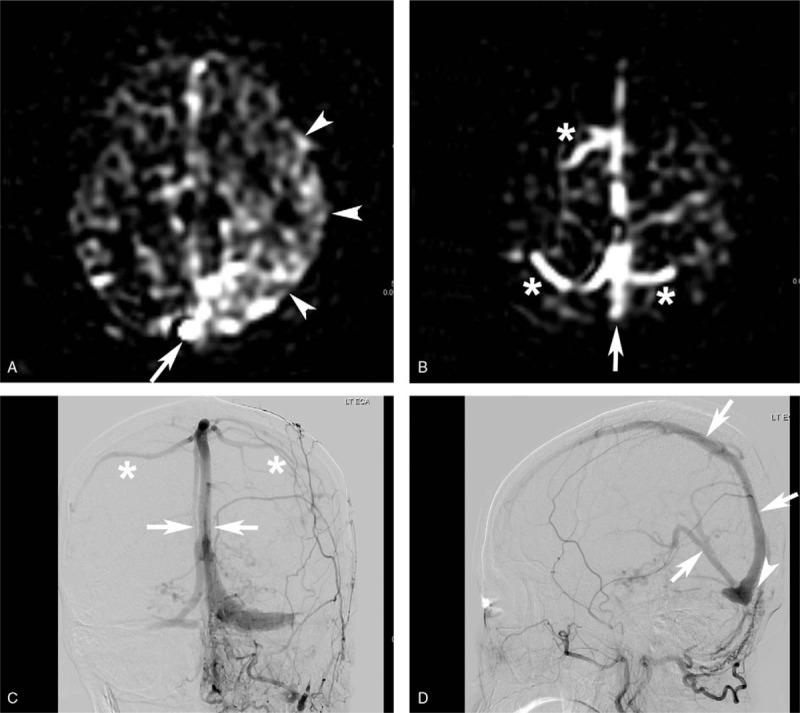
A 55-year-old woman presented with gait disturbance. (A**)** ASL-PWI shows bright signal intensity apparent in the superior sagittal sinus just proximal to torcula (arrow). Bright signal intensities in the brain parenchyma are also shown (arrowheads). (B) ASL-PWI at vertex level shows bright signal intensities in the superior sagittal sinus (arrow) implying retrograde drainage and bright signal intensity in the cortical veins (asterisks) implying presence of CVD. (C and D) DSA images reveal early hypervascular staining in the superior sagittal sinus and straight sinus (arrows) and DAVF at torcula (arrowhead). CVD is also shown on DSA (asterisks). ASL-PWI = arterial spin-labeling perfusion-weighted image, CVD = cortical venous drainage, DAVF = dural arteriovenous fistula, DSA = digital subtraction angiography.

**Figure 3:**
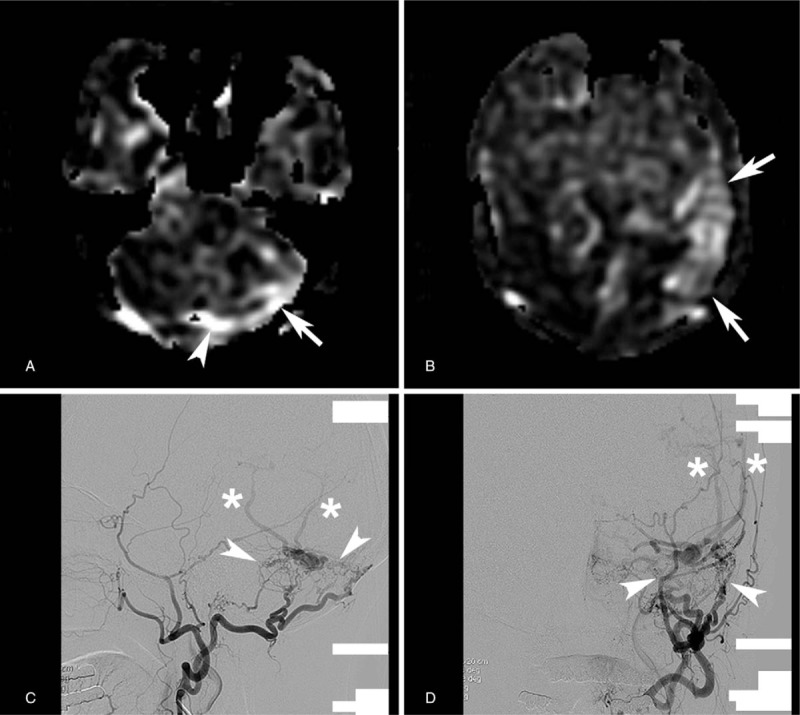
A 79-year-old woman presented with altered mentality. (A) ASL-PWI shows bright signal intensity apparent in the left transverse sinus just proximal to left transverse sinus-sigmoid sinus junction (arrow). Bright signal intensity is also apparent in the left transverse sinus around the midline (arrowhead) implying retrograde draining. (B) ASL-PWI shows bright signal intensities in the brain parenchyma (arrows) implying presence of CVD. However, bright signal intensity in the cortical vein is not definite on ASL-PWI. (C and D) DSA images reveal DAVF at the left transverse sinus-sigmoid sinus junction (arrowheads). CVD is also shown on DSA (asterisks). ASL-PWI = arterial spin-labeling perfusion-weighted image, CVD = cortical venous drainage, DAVF = dural arteriovenous fistula, DSA = digital subtraction angiography.

Representative MR images including ASL-PWI are shown in Figures 1–3.

## 4 Discussion

DAVFs with CVD have an aggressive clinical course. It has been reported that the presence of CVD yields an annual mortality rate of 10.4% with annual risk of intracranial hemorrhage at 8.1% and annual risk of nonhemorrhagic neurologic deficit at 6.9%.^[1]^ Therefore, early treatment such as endovascular intervention or surgery is necessary to avoid complications. On the contrary, patients without CVD have extremely low risk of intracranial hemorrhage.^[14]^ According to Satomi et al,^[15]^ incidence rate of intracranial hemorrhage for DAVFs without CVD was only 1.5% during a mean follow-up of 27.9 months. Regarding treatment, conservative management with close follow-up is indicated for patients without CVD. Thus, detecting CVD in patients with DAVF is clinically important to decide management plans.

ASL-PWI can depict arteriovenous shunting as a high venous ASL-PWI signal intensity due to direct blood flow between feeding arteries and draining veins without intervening capillary bed.^[10–12]^ Visualization of CVD on ASL-PWI images as hyperintensity in cortical veins has been reported in several previous studies.^[10,11]^ Noguchi et al have reported that all 6 patients with DAVF Borden type III and 1 of 4 patients with Borden type II have marked hyperintense areas in cortical veins.^[10,11]^ Amukotuwa et al have reported good diagnostic performance of ASL for detecting CVD.^[10,11]^ Although all patients with DAVF Borden type III demonstrated cortical venous hyperintensity on ASL in both studies, patients with DAVF Borden type II had higher false negative rate of ASL in depicting CVD.^[10,11]^ Unlike previous studies, we hypothesized that blood flow resulting from cortical venous reflux might demonstrate bright signal intensity of affected brain parenchyma on ASL-PWI. As assumed, all patients with CVD (11/11, 100%) showed bright signal intensity in cortical veins and/or bright signal intensity in brain parenchyma on ASL-PWI, showing perfect diagnostic performance for identifying CVD in DAVFs. These results suggest that ASL-PWI could be used as a reliable and noninvasive means to predict the presence of CVD in patients with DAVFs, thus facilitating early diagnosis and treatment strategy planning for DAVFs.

In addition, the present study revealed that all DAVF patients (22/22, 100%) showed bright signal intensity in dural sinuses on ASL-PWI. Although calculation of positive predictive value, negative predictive value, or specificity was impossible due to enrolled population solely consisting of angiographically confirmed DAVF, ASL-PWI might have potential as a completely noninvasive imaging technique to screen patients suspected of DAVF based on neurological symptoms and conventional image. However, in clinical setting, detection of DAVF seems to be a challenge without meticulous and careful interpretation. Using ASL technique, the avid finding of bright signal intensity in dural sinus could aid the detection of DAVF.

Our study had a few limitations. First, the statistical power of this study might have been relatively weak due to its relatively small sample size and inherent limitations of a retrospective study. Second, final diagnoses of DAVF were determined based on DSA. These results might not always coincide with true DAVF. In some patients, images might not detect small DAVF. However, results concerning the presence of CVD would not be affected by small DAVF in the present study. Third, there might be some artifacts in the ASL images. To minimize this problem, we excluded patients with ASL images of poor image quality due to significant artifact. In addition, although visual assessment is not an objective method, we assume that it would be feasible to be used in routine clinical setting.

In conclusion, ASL-PWI could be used as a noninvasive way to predict the presence of CVD in patients with DAVFs. Therefore, it has potential as a screening tool for evaluating DAVF prior to invasive DSA.

## Author contributions

**Conceptualization:** Tae Jin Yun, Roh-Eul Yoo, Koung Mi Kang, Seung Hong Choi, Ji-hoon Kim, Chul-Ho Sohn, Moon Hee Han.

**Data curation:** Ji Hee Kang.

**Formal analysis:** Jong Kook Rhim, Young Dae Cho, Dong Hyun Yoo.

**Supervision:** Tae Jin Yun, Moon Hee Han.

**Writing – original draft:** Ji Hee Kang.

**Writing – review & editing:** Tae Jin Yun.
